# Physicochemical characteristics and consumer acceptance of puddings fortified with *Cudrania tricuspidata* and *Aronia melanocarpa* extracts

**DOI:** 10.1002/fsn3.1790

**Published:** 2020-07-21

**Authors:** Jeong Ho Lee, Il Sook Choi

**Affiliations:** ^1^ Sunchang Research Institute of Health and Longevity Sunchang South Korea; ^2^ Department of Food Science and Nutrition Wonkwang University Iksan South Korea

**Keywords:** aronia *(Aronia melanocarpa)*, consumer acceptance, mandarin melon berry *(Cudrania tricuspidata)*, physicochemical characteristic, pudding

## Abstract

The objectives of the study were to evaluate the physicochemical characteristics of puddings fortified with 0.01% mandarin melon berry (*Cudrania tricuspidata*) and 0%–1.0% aronia (*Aronia melanocarpa*) extracts and to assess the effects of the fortification on consumer acceptance. The soluble solid content of pudding significantly increased as aronia concentration increased, whereas pH levels significantly decreased in a similar concentration‐dependent manner. The texture profiles of hardness, cohesiveness, and chewiness increased significantly in the pudding fortified with 0.01% mandarin melon berry extract compared to those of the control pudding, and these profiles decreased with increasing aronia concentration. One hundred consumers evaluated ten puddings, both with and without acid treatments, in two sessions. Overall acceptance, taste acceptance, and texture acceptance showed no significant differences until 0.1% aronia concentration was reached. However, these differences decreased significantly in the pudding fortified with 0.5% and 1.0% aronia extract. The results demonstrate that the potential application of mandarin melon berry and aronia extract fortification in pudding products should be limited to 0.01% mandarin melon berry and 0.1% aronia concentrations.

## Graphical abstract

This study investigated the potential application of Mandarin melon berry (Cudrania tricuspidata) and Aronia (Aronia melanocarpa) in the pudding preparation. Ten puddings were prepared with 0.01% Mandarin melon berry and 0.0%–1.0% Aronia on conditions with and without acid treatments. The physicochemical characteristics and the consumer acceptance of the berry‐fortified puddings were studied.

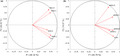

## 1 INTRODUCTION

Mandarin melon berry (*Cudrania tricuspidata*) is a member of the mulberry family and is widespread throughout East Asia. The mandarin melon berry is a common medicinal ingredient in China and Korea due to its bioactive constituents, including polyphenolic compounds, organic acids, and other phytochemicals (Li et al., 2020). Studies have reported antioxidant, anti‐obesity, anti‐inflammatory, anti‐atherosclerotic, and anti‐diabetic properties related to mandarin melon berry (Jo et al., 2017; Kang, Kim, & Youn, 2011; Lin, Lee, Chang, & Yang, 1999; Park et al., 2006; Seo et al., 2000).

Aronia (*Aronia melanocarpa)*, also known as black chokeberry, is a deciduous plant of the family Rosaceae and has been cultivated around North African and Eastern European regions (Wu, Gu, Prior, & McKay, 2004). Aronia is known to have antioxidant, anti‐diabetic, and antiallergic properties, and the use of aronia in food is being emphasized (Jeong, 2008; Kulling & Rawel, 2008; Lee et al., 2013; Li & Jeong, 2015; Malik et al., 2003; Oszmiański & Wojdylo, 2005). Multiple studies have evaluated the effects of aronia fortification on foods such as pork patties, yokan, yogurt dressing, *makgeolli*, and muffin (Chung, 2014; Hwang & Lee, 2013; Kim, Joo, & Choi, 2015; Lee et al., 2014; Park, Kwon, & Moon, 2015).

Although both mandarin melon berry and aronia have gained interest for their multitude of health benefits, these fruits in their raw form have distinctive flavors that can be off‐putting. Mandarin melon berry has a sweet and bland taste, which is related to its high sugar content (16.29 ^o^Brix) and a relatively high pH of 6.5 (Suh, Jung, Lee, & Lee, 2018). Aronia had 16–18 ^o^Brix total soluble solids, containing glucose (30–60 g/L) and fructose (28–58 g/L), and a low pH of 3.6 in freshly pressed juice (Kulling & Rawel, 2008). When consumed raw, aronia is more astringent and has a more sour perception than sweet, due to its tannin concentration (Oszmiański & Wojdylo, 2005). Among the multiple factors that contribute to product acceptance, sensory stimulations, such as taste, smell, and textures, play a vital role (Lawless & Heymann, 2013).

The aims of this study were as follows: (a) determine the physicochemical characteristics of ten puddings prepared with mandarin melon berry extract (0% and 0.01%) and aronia extract (0%, 0.1%, 0.5%, and 1.0%), with and without acid treatment; (b) determine the sensory acceptance level of the puddings fortified with mandarin melon berry and aronia extracts. For this study, pudding was chosen as a food matrix owing to its familiarity among consumers. Commercial puddings have a high sugar content that contributes to their sweet taste and high energy density. Pudding samples in the study had less sugar content than that of commercial puddings, which may have affected the general acceptance levels.

## 2 MATERIALS AND METHODS

### 2.1 Sample preparation

Mandarin melon berry and aronia were supplied by Sunchang Research Institute of Health and Longevity in Sunchang, Jeollabukdo, Korea, in 2016. The mandarin melon berry and aronia were frozen and stored at −20°C until transferred for freeze drying. An extract of the berry was prepared by adding 50 g of berry powder to 1,000 ml of deionized water. The supernatant was further freeze‐dried to obtain freeze‐dried berry extracts. Fructooligosaccharide, gelatin, agar, and low‐fat milk were bought from the local market in Iksan, Jeollabukdo, Korea. Gelatin (1.3%) and agar (0.1%) were used as gelling agents. Fructooligosaccharide (5%) and citric acid (0.1%) were used as a sweetener and acidifier, respectively. In the preliminary experiment, the respondents indicated that the puddings were too astringent and had a hard texture when the mandarin melon berry extract content exceeded 0.01%. Furthermore, the aronia extract content was set from 0.0% to 1.0% due to its strong sourness, astringency, and bitter almond‐like smell when the aronia content exceeded 1.0% in concentration.

The ingredients of the puddings were measured according to the ratio given in Table 1. Pudding samples were prepared based on the method described by Jeong and Kim (2008). Gelatin, Fructooligosaccharide, citric acid, carrageenan, and agar were dissolved in milk before heating up to 60°C. At 60°C, mandarin melon berry and aronia extracts were added and pasteurized for 10 min at 80°C, poured into the pudding mold, and then cooled down at room temperature of 25°C. The pudding samples were stored in the refrigerator at 4°C until physicochemical evaluation.

**TABLE 1:** Formula of pudding preparations fortified with mandarin melon berry (*Cudrania tricuspidata)* and aronia (*Aronia melanocarpa*)

Ingredient (g)	Con^a^	MA0.0	MA0.1	MA0.5	MA1.0	aCon	aMA0.0	aMA0.1	aMA0.5	aMA1.0
Mandarin melon berry	0.0	0.01	0.01	0.01	0.01	0.0	0.01	0.01	0.01	0.01
Aronia	0.0	0.0	0.1	0.5	1.0	0.0	0.0	0.1	0.5	1.0
Sugar	5.0	5.0	5.0	5.0	5.0	5.0	5.0	5.0	5.0	5.0
Acid	0.0	0.0	0.0	0.0	0.0	0.1	0.1	0.1	0.1	0.1
Gelatin	1.3	1.3	1.3	1.3	1.3	1.3	1.3	1.3	1.3	1.3
Carrageenan	0.4	0.4	0.4	0.4	0.4	0.4	0.4	0.4	0.4	0.4
Agar	0.1	0.1	0.1	0.1	0.1	0.1	0.1	0.1	0.1	0.1
Milk	93.2	93.19	93.09	92.69	92.19	93.1	93.09	92.99	92.59	92.09
Total	100	100	100	100	100	100	100	100	100	100

^a^ Con: without berry and without acid; MA0.0:0.01% Mandarin melon berry extract without acid; MA0.1:0.01% Mandarin melon berry extract and 0.1% Aronia extract and without acid; MA0.5:0.01% Mandarin melon berry extract and 0.5% Aronia extract without acid; MA1.0:0.01% Mandarin melon berry extract and 1.0% Aronia extract without acid; aCon: without berry and with acid; aMA0.0:0.01% Mandarin melon berry extract and with acid; aMA0.1:0.01% Mandarin melon berry extract and 0.1% Aronia extract and with acid; aMA0.5:0.01% Mandarin melon berry extract and 0.5% Aronia extract and with acid; aA1.0:0.01% Mandarin melon berry extract and 1.0% Aronia extract and with acid

### 2.2 Determination of physicochemical properties

Total soluble solids of the puddings were quantified by adding deionized water (20 ml) to samples (10 g) and mixing in a shaker ((SHO‐2D, DaiHan Scientific Co.) at the room temperature for 1 hr. Next, the samples were centrifuged (centrifuge 5810f, Eppendorf) at 3,100 *g* for 5 min, and supernatants were collected. The supernatant was then measured using a digital refractometer (N‐1E, Atago, Tokyo, Japan), and the pH was determined using a pH meter (Corning 530, Corning Inc.).

Color values of puddings were examined using a spectrophotometer (Color‐Eye 3,100, Macbeth) tristimulus color analyzer, which was calibrated with a white porcelain reference plate with an L* value of 98.75, a* value of −1.02, and b* value of 1.10. The reflectance and chromaticity determined the color coordinates of the uniform Hunter color space L*, a*, and b*. The L* value indicates the brightness ranging from black (L* = 0) to white (L* = 100). The a* value indicates the redness ranging from green (−60) to red (60), and the b* value indicates the yellowness ranging from blue (−60) to yellow (60).

The texture parameters of hardness, cohesiveness, chewiness, springiness, and adhesiveness of the pudding samples were measured in triplicate with texture profile analysis (Nishinari, Kohyama, Kumagai, Funami, & Bourne, 2013) by utilizing the texture analyzer (Compac‐100 Ⅱ, Scientific). Each pudding was compressed in two cycles, using a cross‐head speed of 1.0 mm/s, to 40% of their original size. This was carried out using a 25 mm diameter cylindrical probe. The compression of the sample in two cycles reflects two human bites and addresses hardness (the required force to compress food at the first bite), representing the peak force of the first cycle. Cohesiveness reflects that the internal structure of a sample withstands compression and is calculated as the ratio of work during compression of the second cycle divided by the first cycle. Chewiness reflects the work required to chew the solid food to a state in which it is ready to be swallowed and is calculated as the multiplication of hardness, cohesiveness, and springiness. Springiness reflects the extent to which compressed food returns to its original size after the load is removed. It is calculated as the ratio of the distances of the time to reach the maximum force in the second compression to the time to reach the maximum force in the first compression. Adhesiveness reflects the work required to overcome the attractive forces between the surface of the food and the surface of other materials, such as teeth, tongue, and palate, and is calculated as the negative area of the first compression.

### 2.3 Consumer acceptance

A total of 102 consumers ranging from 20 to 26 years of age (31 males and 71 females) participated. Two consumers returned insufficient responses and thus have been excluded. Participants were mostly students who were recruited through e‐mail and flyers. The participants were provided with a gift as an incentive for participation. They were asked to refrain from smoking and eating at least 1 hr before the test. The acceptance test was conducted at the sensory laboratory in the human ecology building of Wonkwang University, Iksan, South Korea. The consumers participated in two sessions, which were composed of pudding samples with and without added acid. The first 50 consumers evaluated 5 pudding samples that contained added acid during the first session and evaluated 5 pudding samples without added acid after a 5 min break. The remaining 50 consumers evaluated 5 pudding samples without added acid in the first session and evaluated 5 pudding samples with added acid in the second session, after a 5 min break. They evaluated the samples at am (10:00–11:00 a.m.) or pm (3:00–4:00 a.m.) to avoid lunch hours. Each sample was coded by a different three‐digit number to prevent response bias, and room temperature (20 ± 2°C) water was also provided to rinse the mouth between samples. All samples within each session were presented in the simultaneous presentation manner, in a balanced order, based on the mutually orthogonal latin squares (MOLS) design, which is balanced for k‐1 carryover effects, where k is the number of samples presented to each subject (Wakeling & MacFie, 1995).

Prior to the evaluation, consumers were given instructions regarding evaluation procedures, rinsing method, and rating method and asked to complete an informed consent form. The consumers participated in two sessions, one with acid‐pudding and the other without acid‐pudding, with one break between sessions. In each session, participants evaluated the samples for overall acceptance, followed by acceptance of color, aroma, taste, and texture, using a 9‐point hedonic scale anchored on the left with “dislike extremely” and on the right with “like extremely” (1 = dislike extremely, 9 = like extremely). Consumers were required to expectorate into a spit cup with a cap and rinse their mouth with deionized water before the first sample and between samples. Each session lasted about 20 to 30 min, and the two sessions combined averaged a total of 50 to 60 min.

### 2.4 Statistical analysis

XLSTAT software (2015, Addinsoft) was used for statistical analysis. The data from the physicochemical analyses were analyzed using analysis of variance (ANOVA), with mean separation using Fisher's least significant difference (LSD) at a significance level of 0.05. Ratings of overall acceptance, color acceptance, aroma acceptance, taste acceptance, and texture acceptance of each sample were analyzed by analysis of variance (ANOVA), with mean separation using Fisher's least significant difference (LSD) at a significance level of 0.05. Data visualization was achieved using principal component analysis (PCA).

## 3 RESULTS AND DISCUSSION

### 3.1 Physicochemical properties of pudding

Total soluble solids and pH levels of the puddings fortified with mandarin melon berry and aronia extracts are provided in Table 2. Total soluble solids of the puddings fortified with aronia increased with increase in aronia concentration (MA0.0 = 3.83 ºBx, MA0.1 = 4.00 ºBx, MA0.5 = 4.20 ºBx, MA1.0 = 4.40 ºBx; aMA0 = 3.60 ºBx, aMA0.1 = 3.77 ºBx, aMA0.5 = 3.80 ºBx, aMA1.0 = 3.90 ºBx). Skupień and Oszmiański (2007) have shown that soluble solids of aronia fruits were 24.1 ºBx, and reducing sugar was 19.35 g/100 g FW. Kulling and Rawel (2008) have also shown that the content of reducing sugar in fresh aronia berries was between 16%–18% and the sum of glucose and fructose was between 13–17.6 g/100g FW. The pH of the pudding fortified with mandarin melon berry and aronia decreased as aronia concentration increased. It has been shown that the total content (1%–1.5% of FW) of organic acid in aronia is relatively low when compared to other berries, and the main organic acids identified are citric acid and malic acid, with pH 3.3–3.9 (Kulling & Rawel, 2008).

**TABLE 2:** Physicochemical Properties and texture profiles of puddings fortified with mandarin melon berry (*Cudrania tricuspidate*) and Aronia (*Aronia melanocarpa*) extracts without acid treatment and with acid treatment

Pudding^a^	Physicochemical Properties					Texture Profiles				
	Total soluble solids (^o^Bx)	pH	L* value	a* value	b* value	Hardness (*N*)	Cohesiveness (−)	Chewiness (N*mm)	Springiness (m)	Adhesiveness (J)
Con	3.83 ± 0.06*^de^*	6.68 ± 0.01*^ab^*	75.82 ± 0.12*^a^*	−1.17 ± 0.01*^hr^*	2.16 ± 0.08*^cd^*	262.67 ± 3.51*^f^*	53.48 ± 0.26*^f^*	139.30 ± 2.95*^g^*	163.13 ± 1.67*^b^*	−27.33 ± 1.15*^a^*
MA0.0	3.83 ± 0.06*^de^*	6.69 ± 0.01*^a^*	75.67 ± 0.15*^a^*	−1.16 ± 0.13*^gh^*	2.54 ± 0.05*^c^*	428.33 ± 2.52*^a^*	65.31 ± 0.59*^bcd^*	280.82 ± 2.99*^a^*	101.52 ± 0.16*^de^*	−34.67 ± 0.58*^bc^*
MA0.1	4.00 ± 0.00*^e^*	6.66 ± 0.01*^b^*	73.76 ± 0.03*^d^*	−0.43 ± 0.01*^f^*	2.05 ± 0.26*^d^*	281.67 ± 6.11*^e^*	56.49 ± 1.05*^ef^*	154.56 ± 0.35*^f^*	101.12 ± 0.19*^de^*	−28.67 ± 2.52*^ab^*
MA0.5	4.20 ± 0.00*^b^*	6.56 ± 0.02*^c^*	69.08 ± 0.20*^f^*	1.22 ± 0.06*^d^*	0.93 ± 0.31*^e^*	334.67 ± 1.53*^c^*	58.34 ± 2.46*^def^*	194.07 ± 3.58*^d^*	100.89 ± 0.19*^de^*	−35.00 ± 1.73*^c^*
MA1.0	4.40 ± 0.00*^a^*	6.45 ± 0.02*^d^*	64.53 ± 0.14*^hr^*	2.30 ± 0.03*^b^*	0.57 ± 0.10*^ef^*	381.00 ± 4.58*^b^*	62.14 ± 1.66*^bcde^*	203.58 ± 5.37*^c^*	100.56 ± 0.19*^e^*	−37.33 ± 4.04*^cd^*
aCon	3.53 ± 0.06*^f^*	6.11 ± 0.01*^e^*	74.37 ± 0.46*^c^*	−1.14 ± 0.02*^gh^*	2.96 ± 0.18*^ab^*	295.33 ± 4.73*^d^*	68.79 ± 0.61*^ab^*	211.12 ± 1.83*^cd^*	175.64 ± 1.45*^a^*	−45.00 ± 1.73*^e^*
aMA0.0	3.60 ± 0.00*^f^*	6.12 ± 0.01*^ef^*	75.10 ± 0.11*^b^*	−1.00 ± 0.00*^g^*	3.22 ± 0.22*^a^*	375.33 ± 4.04*^b^*	74.35 ± 6.83*^a^*	260.80 ± 5.70*^b^*	101.47 ± 0.52*^de^*	−42.33 ± 0.58*^de^*
aMA0.1	3.77 ± 0.06*^e^*	6.08 ± 0.01*^f^*	71.92 ± 0.02*^e^*	0.19 ± 0.04*^e^*	2.16 ± 0.16*^cd^*	276.00 ± 1.00*^e^*	65.73 ± 0.23*^bc^*	175.22 ± 4.31*^e^*	103.68 ± 0.90*^cd^*	−38.00 ± 1.00*^cd^*
aMA0.5	3.80 ± 0.00*^de^*	5.99 ± 0.01*^g^*	67.23 ± 0.04*^g^*	1.96 ± 0.10*^c^*	0.95 ± 0.08*^e^*	250.33 ± 6.43*^g^*	60.90 ± 0.39*^cde^*	155.30 ± 7.55*^f^*	105.27 ± 1.59*^c^*	−32.67 ± 1.15*^abc^*
aMA1.0	3.90 ± 0.00*^cd^*	5.86 ± 0.02*^hr^*	63.17 ± 0.14*^i^*	3.20 ± 0.06*^a^*	0.39 ± 0.06*^f^*	165.00 ± 2.00*^hr^*	51.39 ± 2.75*^f^*	83.32 ± 6.27*^hr^*	106.57 ± 0.85*^c^*	−28.00 ± 3.61*^a^*

Note

The same letters present not significantly different values determined by the least significant difference (LSD) mean separation test between sample group.

^a^ Con: without berry and without acid; MA0.0:0.01% mandarin melon berry extract without acid; MA0.1:0.01% mandarin melon berry extract and 0.1% aronia extract and without acid; MA0.5:0.01% mandarin melon berry extract and 0.5% aronia extract without acid; MA1.0:0.01% mandarin melon berry extract and 1.0% aronia extract without acid; aCon: without berry and with acid; aMA0.0:0.01% mandarin melon berry extract and with acid; aMA0.1:0.01% mandarin melon berry extract and 0.1% aronia extract and with acid; aMA0.5:0.01% mandarin melon berry extract and 0.5% aronia extract and with acid; aA1.0:0.01% mandarin melon berry extract and 1.0% aronia extract and with acid

The color parameters of pudding fortified with different concentrations of mandarin melon berry and aronia extracts are shown in Table 2. The L* value and b* value of pudding with acid treatment decreased with increasing concentration of aronia extract, while the a* value of pudding increased. Con (control pudding without acid) and aCon (control pudding with acid) without mandarin melon berry and aronia fortifications had the lowest a* values of −1.17 and −1.14, respectively. In contrast, MA1.0 (pudding with 0.01% mandarin melon berry and 1.0% aronia) and aMA1.0 (acid treated pudding with 0.01% mandarin melon berry and 1.0% aronia) had the highest level of a* values of 2.30 and 3.20, respectively. Denev Kratchanov Ciz Lojek and Kratchanova (2012) reviewed the antioxidant activities of aronia in respect of its major anthocyanins, such as cyanidin‐3‐arabinoside (94 to 582 mg/100 g), cyanidin‐3‐galactoside (101 to 1,282 mg/100 g), cyanidin‐3‐glucoside (1.7 to 38 mg/100 g), and pelargonidin‐3‐arabinoside (2.3 mg/100 g). The quinoidal blue species are predominant in acidic conditions at pH 2 to 4; meanwhile, four structural forms of anthocyanins, such as red flavylium cations, colorless carbinol base, pale yellow chalcone, and anhydrous quinoidal base, have been observed at pH 4–6 (Castaneda‐Ovando, Pacheco‐Hernández, Páez‐Hernández, Rodríguez, & Galán‐Vidal, 2009). Under more acidic conditions, the amount of red flavylium cations increases, but when the pH increases the amount of pale yellow chalcone also increase (Castaneda‐Ovando et al., 2009). In this study, a* values (redness) of each sample increased both as aronia concentrations increased and with acid treatments to the puddings. The b* values (yellowness) of each sample decreased with increasing aronia concentrations, related to the increase of acidic conditions. The b* values were at their highest in puddings fortified with mandarin melon berry only (MA0.0, and aMA0.0), whereas the pudding fortified with 1.0% aronia with acid (aMA1.0) had the lowest b* values and pH (pH 5.86). This pattern is in accordance with the findings of the study by Hwang and Lee (2013), who reported significant decreases in L* values and b* values, as well as increases in a* values, with the increase of anthocyanin concentration in foods.

The texture profiles of the puddings fortified with mandarin melon berry and aronia are shown in Table 2. In both the puddings with and without acid treatment, hardness, cohesiveness, and chewiness significantly increased in the puddings treated with 0.01% mandarin melon berry only (MA0.0 and aMA0.0) when compared to the control puddings (Con and aCon). A study by Jung Ju Choi You and Noh (2013) has reported that mandarin melon berry had 9.3 mg% of Fe, which is higher than 0.4 mg% of Fe in aronia (Kulling & Rawel, 2008). When added to milk, iron reacts with organic and inorganic phosphates of casein micelles and causes an increase in aggregation (Broyard & Gaucheron, 2015). In this study, one of the ingredients of pudding preparation was milk, and mandarin melon berry may therefore have reacted with the casein micelles in milk and led to increased values for hardness, cohesiveness, and chewiness. In the puddings without acid treatments (Con, MA0.0, MA0.1, MA0.5, MA1.0), hardness, chewiness, and adhesiveness were significantly higher than the puddings with acid treatment (aCon, aMA0.0, aMA0.1, aMA0.5, aMA1.0). Furthermore, hardness, cohesiveness, chewiness, and adhesiveness decreased in a concentration‐dependent manner as aronia concentrations increased (*p* < .05). Another ingredient used in pudding preparation was gelatin (1.3%) for improving texture profiles. Gelling of gelatin is correlated to the temperature, sugar content, and pH level (Djagny, Wang, & Xu, 2001; Gioffre, Torricelli, Panzavolta, Rubini, & Bigi, 2012). The firmness of gelatin gels with milk protein was influenced by pH (Pang, Deeth, Sopade, Sharma, & Bansal, 2014). Li and Gu (2011) has shown that particles of gelatin solution with pH less than 6.0 had small particle sizes. However, fast increases of particle size were observed in pH 6.0 and above, results which are related to weak zeta‐potential and a decrease of repulsion among particles. In this study, the pH values decreased from 6.68 (Con) to 6.45 (MA1.0) in puddings without acid treatment as aronia concentration increased. In comparison, the pH values decreased from 6.11 (aCon) to 5.86 (aMA1.0) in puddings with acid treatment. The decreasing pH in puddings with acid treatments might have resulted in the decreasing texture profiles when compared with puddings without acid treatment.

### 3.2 Consumer acceptability

**FIGURE 1:**
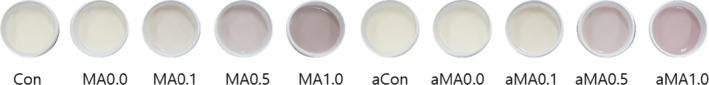
Photograph of the pudding fortified with mandarin melon berry (*Cudrania tricuspidata*) and aronia (*Aronia melanocarpa*) without and with acid treatment

The mean scores of 100 consumers’ ratings of overall acceptance and acceptances of color, aroma, taste, and texture for puddings fortified with mandarin melon berry and aronia with and without acid treatments are shown in Table 3. There were significant differences in overall, taste, and texture acceptance for pudding samples both without (Con, MA0.0, MA0.1, MA0.5, MA1.0) and with acid treatments (aCon, aMA0.0, aMA0.1, aMA0.5, aMA1.0), with aroma acceptance being the exception to this trend. The scores of color acceptance significantly increased in puddings fortified with mandarin melon berry and aronia in concentration‐dependent manners. In contrast, the scores of overall acceptance significantly decreased in the puddings with more than 0.5% aronia concentration (MA0.5, MA1.0, aMA0.5, and aMA1.0) compared to the puddings with less than 0.5% aronia concentration (Con, MA0.0, MA0.1, aCon, aMA0.0, and aMA0.1). The pudding group with acid had slightly higher mean scores compared to those of the pudding group without acid, under the same concentration conditions, but the difference was not significant. For the puddings fortified with mandarin melon berry and aronia, a high concentration of aronia (MA0.5, aMA0.5, MA1.0, aMA1.0) led to a significant decrease in both overall and texture acceptance, similar to the patterns seen in the texture analysis data. Furthermore, although the puddings fortified only with mandarin melon berry (MA0.0, aMA0.0) showed significantly higher values for hardness and cohesiveness in the texture analysis, they did not influence the consumer acceptance test as significantly as the addition of aronia concentration did (Figure 1).

**TABLE 3:** Mean scores for consumer acceptability of the puddings fortified with mandarin melon berry (*Cudrania tricuspidata*) and aronia (*Aronia melanocarpa*) extracts without and with acid treatment

Pudding^a^	Overall acceptance	Color acceptance	Aroma acceptance	Taste acceptance	Texture acceptance
Con	6.2 ± 1.7^a^	4.6 ± 1.3^e^	5.2 ± 1.6^a^	6.0 ± 2.1^a^	6.1 ± 1.8^ab^
MA0.0	5.9 ± 1.7^ab^	5.2 ± 1.3^bcd^	5.4 ± 1.5^a^	5.7 ± 1.4^a^	6.0 ± 1.5^ab^
MA0.1	6.0 ± 1.4^a^	5.4 ± 1.1^abc^	5.4 ± 2.2^a^	5.5 ± 1.5^ab^	5.9 ± 1.7^abc^
MA0.5	5.4 ± 1.5^c^	5.6 ± 1.2^ab^	5.5 ± 1.3^a^	5.2 ± 1.5^bc^	5.6 ± 1.7^bcd^
MA1.0	4.9 ± 1.7^d^	5.8 ± 1.9^a^	5.6 ± 1.7^a^	4.9 ± 1.6^c^	5.3 ± 1.6^d^
aCon	6.2 ± 1.7^a^	4.8 ± 2.1^de^	5.3 ± 1.4^a^	6.0 ± 1.8^a^	6.1 ± 1.8^a^
aMA0.0	6.0 ± 1.6^a^	5.1 ± 2.1^cd^	5.3 ± 2.0^a^	5.8 ± 1.8^a^	6.2 ± 2.1^a^
aMA0.1	6.1 ± 1.5^a^	5.4 ± 1.7^bc^	5.4 ± 1.4^a^	5.7 ± 2.0^a^	5.9 ± 1.6^abc^
aMA0.5	5.5 ± 1.5^bc^	5.6 ± 1.8^ab^	5.5 ± 1.7^a^	5.5 ± 1.9^ab^	5.5 ± 1.4^cd^
aMA1.0	4.9 ± 1.8^d^	5.7 ± 1.6^ab^	5.6 ± 1.8^a^	5.2 ± 1.8^bc^	5.2 ± 1.8^d^

Note

The same letters present not significantly different values determined by the least significant difference (LSD) mean separation test between sample group.

**FIGURE 2:**
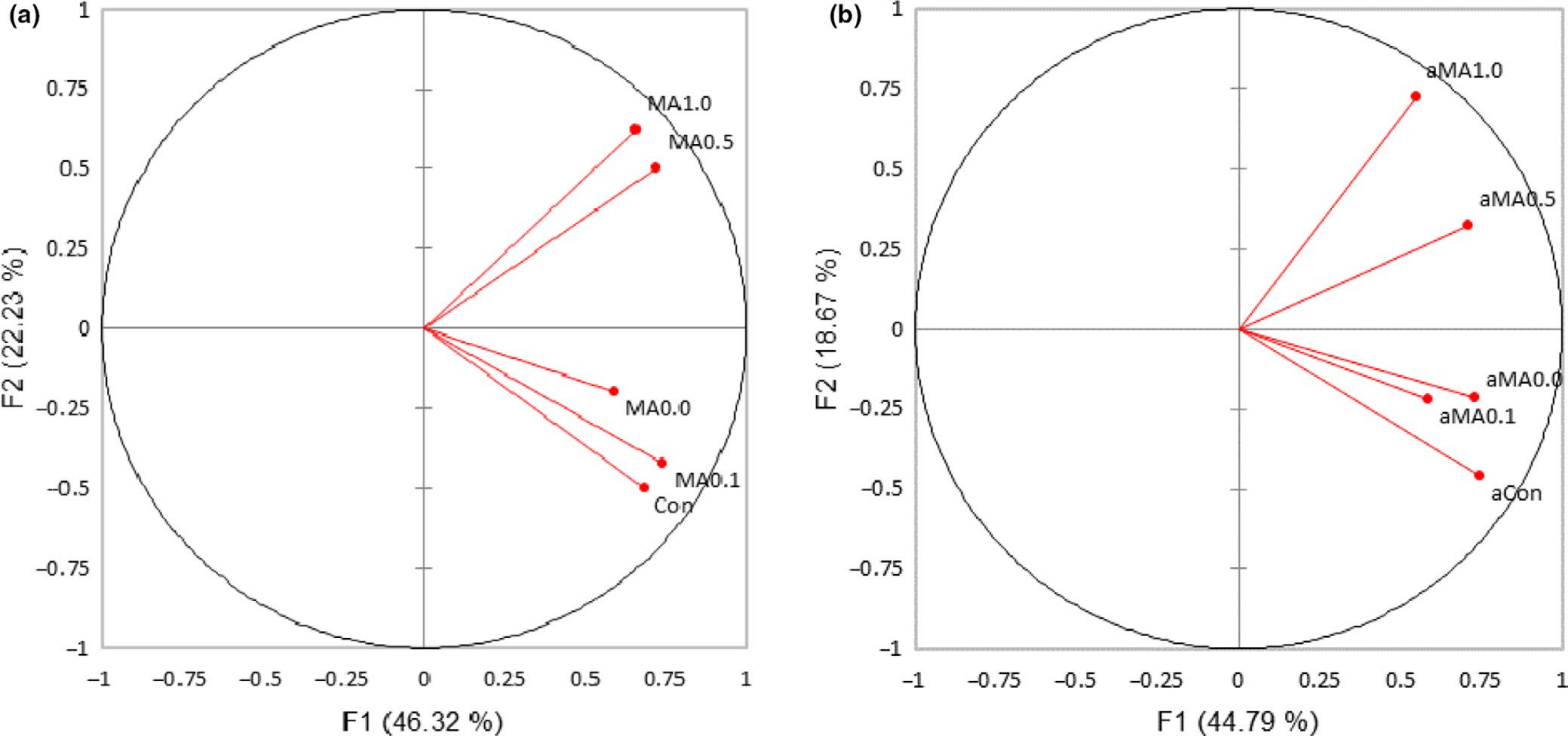
Principal component analysis (PCA) biplot of overall acceptance ratings by 100 consumers for puddings fortified with mandarin melon berry (*Cudrania tricuspidata*) and aronia (*Aronia melanocarpa*) extracts without (a) and with acid treatment (b)

To examine the consumer acceptance patterns toward the pudding samples, overall acceptance ratings were visualized using PCA (Figure 2). From Figure 2a, the PCA biplot represented 68.54% of the total variation (F1 = 46.32% and F2 = 22.23%). All puddings were located on the positive side of the F1‐axis, but only MA1.0 and MA0.5 were located on the positive side of the F2‐axis, both of which obtained lower overall acceptance scores than those of other puddings (MA0.0, MA0.1, Con). From Figure 2b, the PCA biplot represented 63.47% of the total variation (F1 = 44.79% and F2 = 18.67%). All puddings were located on the positive side of the F1‐axis, but the puddings fortified with low concentration of aronia (MA0.0, MA0.1, Con) were located on the negative side of the F2 axis. The PCA results of pudding with acid treatment had shown similar patterns to those of puddings without acid treatment.

The limitation of this study is that the consumer acceptance questions did not determine the sensory intensities, such as sweetness, sourness, and smoothness, to clarify their correlation with physicochemical characteristics. Therefore, future consumer acceptance, including attribute intensity with descriptive analysis, would be helpful to determine attribute characteristics with intensities of products containing berries.

## 4 CONCLUSIONS

This study evaluated the physicochemical properties and consumer acceptance of ten puddings fortified with varying concentrations of mandarin melon berry and aronia extracts, under conditions with and without acid treatment. Texture profiles of hardness, cohesiveness, chewiness, and adhesiveness significantly increased in the puddings fortified with 0.01% mandarin melon berry (MA0.0 and aMA0.0) and decreased with increasing aronia concentration. The consumer acceptance scores showed no significant differences in the puddings for Con, MA0.0, and MA0.1 samples. Overall, taste and texture acceptances decreased in MA0.5 and MA1.0, while color acceptance increased as aronia concentrations increased. Similar patterns were also observed in the puddings treated with acid. The results indicate that the application of mandarin melon berry and aronia extract into pudding products should be limited to 0.01% mandarin melon berry and 0.1% aronia concentrations to prevent a significant decrease in overall acceptance. One of the limitations of the study is that there were no significant differences between the texture and consumer acceptance test results of MA0.0 and aMA0.0, despite the significant changes in values of texture analysis. Other limitations of the study include that the sensory discussions did not clarify the correlation of sensory intensities with physicochemical characteristics. Therefore, further studies could be useful in determining optimal mandarin melon berry and aronia concentrations and the correlation between consumer acceptance and sensory intensities.

## CONFLICT OF INTEREST

There is no potential conflict of interest reported by the authors.

## ETHICAL APPROVAL

This study was approved by Wonkwang Institutional Review Board.

## INFORMED CONSENT

This study obtained written informed consent from all study participants.
